# Investigating Homology between Proteins using Energetic Profiles

**DOI:** 10.1371/journal.pcbi.1000722

**Published:** 2010-03-26

**Authors:** James O. Wrabl, Vincent J. Hilser

**Affiliations:** 1 Department of Biochemistry and Molecular Biology, University of Texas Medical Branch at Galveston, Galveston, Texas, United States of America; 2 Sealy Center for Structural Biology and Molecular Biophysics, University of Texas Medical Branch at Galveston, Galveston, Texas, United States of America; Stanford University, United States of America

## Abstract

Accumulated experimental observations demonstrate that protein stability is often preserved upon conservative point mutation. In contrast, less is known about the effects of large sequence or structure changes on the stability of a particular fold. Almost completely unknown is the degree to which stability of different regions of a protein is generally preserved throughout evolution. In this work, these questions are addressed through thermodynamic analysis of a large representative sample of protein fold space based on remote, yet accepted, homology. More than 3,000 proteins were computationally analyzed using the structural-thermodynamic algorithm COREX/BEST. Estimated position-specific stability (*i.e*., local Gibbs free energy of folding) and its component enthalpy and entropy were quantitatively compared between all proteins in the sample according to all-*vs.*-all pairwise structural alignment. It was discovered that the local stabilities of homologous pairs were significantly more correlated than those of non-homologous pairs, indicating that local stability was indeed generally conserved throughout evolution. However, the position-specific enthalpy and entropy underlying stability were less correlated, suggesting that the overall regional stability of a protein was more important than the thermodynamic mechanism utilized to achieve that stability. Finally, two different types of statistically exceptional evolutionary structure-thermodynamic relationships were noted. First, many homologous proteins contained regions of similar thermodynamics despite localized structure change, suggesting a thermodynamic mechanism enabling evolutionary fold change. Second, some homologous proteins with extremely similar structures nonetheless exhibited different local stabilities, a phenomenon previously observed experimentally in this laboratory. These two observations, in conjunction with the principal conclusion that homologous proteins generally conserved local stability, may provide guidance for a future thermodynamically informed classification of protein homology.

## Author Summary

Protein structure and function are fundamentally determined by thermodynamics. However, for technical as well as historical reasons, current evolutionary classification schemes and bioinformatics tools do not fully utilize thermodynamic information to describe or analyze proteins. In this work, we address this deficiency by computationally estimating the position-specific thermodynamic quantities of stability (ΔG), enthalpy (ΔH), and entropy (TΔS) for a large and diverse representative sample of protein structures. The sample was drawn from an expertly curated database, such that accepted evolutionary relationships existed for all protein pairs. Importantly, trivial relationships between pairs highly similar in amino acid sequence were explicitly excluded. We found that all position-specific thermodynamic quantities ΔG, ΔH, and TΔS were more similar between proteins that were evolutionarily related (*i.e.,* homologous), and were less similar between proteins that were not evolutionarily related (*i.e.,* non-homologous), with stability being particularly similar between homologous proteins. However, interesting statistically significant exceptions to these trends were observed, exceptions that could indicate novel processes of functional adaptation or evolutionary fold change, mediated by thermodynamics, for the proteins involved. Taken together, these results expand our understanding of the role of thermodynamics in protein evolution and suggest an organizational framework for a future thermodynamically-informed classification of protein homology.

## Introduction

Protein structure and function are ultimately determined by thermodynamics. For example, Anfinsen's seminal work [1] demonstrated that the native state of a protein exists at a minimum in Gibbs free energy of stability under physiological conditions. Binding and catalysis are also governed by free energy: the sign and magnitude of the free energy change of each functional reaction controls the reaction's direction and equilibrium extent, respectively [2],[3].

Gibbs free energy (ΔG) results from the summed, often opposing, contributions of enthalpy (ΔH) and entropy (TΔS): ΔG = ΔH−TΔS. Generally, in the case of proteins, changes in free energy are small as compared to the underlying enthalpic or entropic changes [4]. Reactions can be dominated by either enthalpy or entropy, but it is most often the case that a sometimes delicate balance between enthalpy and entropy controls protein structure and function.

Unfortunately for the goal of thermodynamic characterization of protein folds, each of these quantities can be challenging to accurately predict. While enthalpy can be rationalized in terms of information derived from atomic coordinates (*i.e.* from the number and types of bonds seen in the structure) [5], entropy is harder to estimate, frequently requiring knowledge not apparent from a single structure, such as information about the conformational degeneracy of the protein [6]–[8]. Equally as challenging is the task of developing a robust analysis that reports the position-specific (*i.e.* local) stability within the protein, rather than reporting either: 1) the energetic contribution of a residue (which would be highly sequence-dependent) or 2) the stability of a protein as a whole (*i.e.* global stability).

Due in part to the inherent difficulty of accurately computing global and local enthalpy, entropy, and free energy, all protein structure classification strategies of which we are aware do not incorporate thermodynamic information. It is our hypothesis that this theoretical omission limits the complete understanding of protein fold space. There may also be practical consequences to such an omission. For example, it is possible that thermodynamic information, as a protein observable independent of sequence or structure[9], could improve computational tools for sequence alignment, fold recognition [10], or homology detection, thereby clarifying discrepancies in existing classification schemes that are based on only sequence and structure.

Thermodynamic information may also yield new understanding, not available from current schemes, about evolutionary sequence, structure, and functional relationships [11]. One particularly important and as yet unanswered question is the degree to which protein stability and its components (*i.e.* enthalpy and entropy) are conserved during fold evolution: does the concept of “thermodynamic homology” meaningfully exist beyond conservative point mutations?

As a step towards integration of thermodynamic information into existing protein classification schemes, the local (or position-specific) free energy of stability (ΔG), enthalpy (ΔH), and entropy (TΔS) are here computed for a large representative database of protein domains using the previously described COREX/BEST algorithm [12]–[14]. Importantly, the diverse proteins studied have accepted evolutionary relationships [15] and are expertly curated [16] such that any homologs are remote (*i.e.* “twilight zone” [17] pairwise sequence identity or less on average). Thus, by experimental design, trivial comparisons between the thermodynamics of closely related proteins are explicitly excluded from this analysis. The central aim of this work is to assess the degree of thermodynamic conservation among remotely homologous protein domains.

Three findings relating thermodynamics to protein sequence and structure are reported. First, in accordance with previous work [18], it is confirmed that homologous proteins exhibit correlated thermodynamic information. Second, enthalpy and entropy are less correlated than stability, suggesting that homologous sequence differences result in enthalpic and entropic changes that largely balance to preserve the local stability of an evolved protein as compared to an ancestral one. Third, based on manual inspection of structural and thermodynamic alignments of homologous and non-homologous pairs of proteins, an organizational framework is postulated to guide the future integration of COREX/BEST thermodynamic information into theories of protein fold evolution.

## Materials and Methods

### Selection and processing of protein structure data

Structural coordinates for all protein domains of length less than or equal to 150 residues were obtained from the ASTRAL 1.69 database [16] of 40% maximum sequence identity representatives. Those domains defined as SCOP [15] class “e” (membrane protein domains) were discarded, as the COREX/BEST algorithm was parameterized for globular proteins and thus was not expected to accurately estimate the thermodynamic characteristics of membrane proteins. To focus on single domains, those included in SCOP class “f” (multidomain proteins) were also discarded.

Coordinate files were preprocessed and standardized to minimize run-time errors during subsequent analysis; these minor edits included modification of selenomethionine residues to methionine, removal of multiple atom occupancies other than “A”, removal of multiple NMR models other than “1”, and modification of non-standard amino acids to alanine. In total, 3,688 domains from 666 unique SCOP families, 463 SCOP superfamilies, and 292 SCOP folds were represented within the five SCOP classes: all-α, all-β, α+β, α/β and small proteins. These statistics demonstrated a reasonable and diverse sampling of single domain protein structure space, yet included thousands of homologous protein pairs (as defined by SCOP) at less than approximately “twilight-zone” (*i.e.* <25%) sequence identity.

### Computation of local thermodynamic stability, enthalpy, and entropy using the COREX/BEST algorithm

The COREX/BEST algorithm [12]–[14] constructs a protein conformational ensemble using its high-resolution structure as a template. COREX/BEST requires as input the three-dimensional structural coordinates of a protein and employs a sliding window to generate a large number of conformational microstates varying from fully folded to fully unfolded. Output is a thermodynamic (*i.e.* energetic) model of the protein's native state ensemble. The algorithm has been tested by both retrospective validation and blind prediction [12], [14], [19]–[23], and thus has been demonstrated to reasonably represent the ensemble. For this work, a COREX/BEST analysis was performed on each member of the preprocessed ASTRAL database described above using standard parameters: window size, 12; minimum window size, 4; temperature *T*, 25.0°C; and entropy weighting, *W*, 0.5.

The strength of COREX/BEST is the ability to capture local, also known as “position-specific”, thermodynamic quantities. The important distinguishing feature of these position-specific quantities is that they reflect the ensemble-averaged thermodynamic contributions of *many* residues in the three-dimensional neighborhood of one residue, rather than reflecting the independent contribution of only that particular residue [24]. Thus, local thermodynamic quantities, although reported at individual residue positions, greatly depend on the rest of the protein, in the sense that surrounding residues may influence the probability of a particular residue being folded, making it more likely, for example, for blocks of folded residues to be found together. In other words, this ensemble-based formalism separates the energetic contribution of the residue from the position itself. It is possible, and preferable, for these quantities to be obtained from experiment, for example local stability as measured by NMR-detected hydrogen exchange[25] or local enthalpy as measured by the temperature dependence of local stability [26]–[28]. Indeed, comparisons with such experiments have shown that COREX/BEST thermodynamic quantities plausibly reproduce the measured values [12],[14]. However, large scale studies such as the present one are currently difficult, if not impossible, to execute experimentally.

Computation of position-specific thermodynamic quantities from a COREX/BEST ensemble has been described in detail [12],[24],[29]. Briefly, for each partially folded microstate *i* of the ensemble, a Gibbs free energy of global stability ΔG*_i_* is computed from a previously validated and calibrated energy function composed of solvent-exposed surface area and conformational entropy terms [12]. From these stabilities, the probability P*_i_* of each microstate *i* can be estimated by
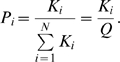
(1)


In Equation (1), *K_i_* = exp(−ΔG*_i_*/RT) is the statistical weight of each microstate, R is the gas constant and Q is the partition function for the system. Given the probabilities of each microstate, a so-called “residue stability constant”, κ*_f,j_*, can be defined for every residue *j* of the protein [12]:
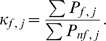
(2)


In Equation (2), the numerator is the summed probability of states in the ensemble in which a particular residue *j* is in a folded conformation and the denominator is the corresponding sum for states in which residue *j* is in an unfolded conformation. The residue stability constant directly gives the local thermodynamic stability ΔG at each residue position *j*, equivalent to the difference in energy between the Boltzmann-weighted subensembles of states in which residue *j* is folded (*f*) and unfolded (*nf*) [24],[29]:
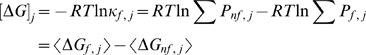
(3)


Similarly, local enthalpy (ΔH) and entropy (TΔS) were computed as a function of residue position *j* in each protein from the COREX/BEST ensembles as differences between the folded and unfolded subensembles for each respective thermodynamic descriptor [24]:
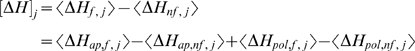
(4)

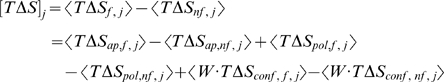
(5)


In Equations (4) – (5), subscript “*ap*” refers to energetic contributions arising from apolar solvent accessible surface area, “*pol*” refers to contributions from polar surface area, and “*conf*” refers to conformational entropy. The specific values of *T* and *W* are given above. Note that the total entropy of the calculation, Equation (5), reflects contributions from both solvent and conformational terms, while the enthalpy, Equation (4), reflects contributions from only solvent. Thus, this statistical thermodynamic treatment can distinguish between the two main classes of entropy. Under the native state conditions simulated in this work, the total entropy appears largely dominated by solvent contributions (Text S1, Figure S1.).

### Thermodynamic pairwise residue equivalences obtained from structural alignment

At least two different strategies could be envisioned to compare local thermodynamic quantities of two proteins: direct alignment of thermodynamic quantities or alignment of quantities according to residue equivalencies obtained from another source. Although the former strategy is under development [18],[30], for expediency we chose here to implement the latter strategy by aligning thermodynamic quantities according to structure alignment. Pairwise structure alignment was performed for the proteins in the dataset in an all-*vs.*-all manner using the DALI-Lite package [31] with default parameters. More than 6 million nonredundant pairwise comparisons were attempted; approximately 95% of these comparisons were successful and were retained for further analysis.

### Quantitative correlation of structurally equivalenced thermodynamic descriptors

Given two sets of *N* equivalenced thermodynamic descriptors, a Pearson correlation coefficient *r* [32] was computed using the equation:
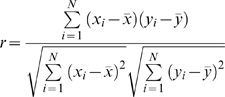
(6)where, *x* and *y* represent sets, one set from each protein, of thermodynamic descriptors (ΔG, ΔH, or TΔS from Equations (3) – (5), the corresponding correlation coefficients are denoted *r_ΔG_*, *r_ΔH_*, *r_TΔS_*, respectively, in the text). The horizontal bar indicates an average.

A perfect positive correspondence was given by *r* = +1, no correspondence by *r* = 0, and a perfect negative correspondence by *r* = −1. Structural alignments of less than an arbitrary length cutoff of 20 residues were ignored, to reduce artifactual correlations due to the sensitivity of the Pearson *r* to outlier data points. Thermodynamic descriptors of the first or last four residues in every protein were also ignored, due to end effects in the COREX/BEST calculation caused by the minimum window size.

The Spearman rank-order correlation method [32], perhaps less widely used but more statistically rigorous than the Pearson *r*, was implemented as an additional test of the robustness of the results. It was observed in essentially all pairwise thermodynamic comparisons, regardless of homology, that the Spearman and Pearson *r* values were highly correlated (Pearson *r* = 0.92, Pearson *p*<10^−6^, Spearman *r* = 0.92, Spearman *p*<10^−6^, 9,241,311 points, data not shown), with significant individual Spearman *p*-values of *p*<0.05 occurring at Pearson *r* values of approximately |*r*|>0.25. As this threshold value of significance represented more than 45% of all 9,241,311 data points, it was decided to report the data in terms of the more widely used Pearson *r*. However, it is emphasized that the qualitative results and conclusions drawn were unchanged whether the Pearson or Spearman methods were used.

A relatively small, but not necessarily exhaustive, number (<50) of homologous protein comparisons involving conformational changes (data not shown) were discovered through manual inspection and discarded, since the conformational change usually dominated the thermodynamics. Although biologically interesting and deserving of future investigation, these changes were not the principal objects of the present study. Mode estimations for probability distributions of correlation coefficients and other quantities were computed using the method of Bickel and Fruewirth [33]. The results reported below were additionally filtered to only include relatively well-determined X-ray crystallographic structures (resolution of ≤2.5 Å). However, all conclusions were unchanged when NMR structures and structures with resolution >2.5 Å were also included (data not shown).

### Null models to estimate significance of correlated thermodynamic descriptors

The statistical significance of individual structural and thermodynamic alignments was assessed through construction of two simple null models. In Null Model 1, the probability of chance occurrence at a particular level of structural or thermodynamic similarity was empirically estimated from the frequency of observed length-matched DALI-alignments at or above the particular similarity level. In this model, separate background distributions were used for homologs and non-homologs. In Null Model 2, the probability of chance occurrence at a particular level of structural or thermodynamic similarity was estimated from the frequency of observed length-matched gapless alignments between randomly selected pairs of non-homologous protein fragments. In this model, a minimum alpha-carbon RMSD structure superposition [34]–[36] of the fragment pair as well as the Pearson *r*-value between thermodynamic descriptors was computed. 30,000 pairs of fragments were chosen for each gapless alignment length *L*, where 10≤*L*≤100. In effect, the two null models occupied extremes of background distributions: Null Model 1 accounted for the interdependence of thermodynamic and structural similarity, while Null Model 2 weakened this interdependence. In both models, *p*-values were conservatively estimated, rounding up to the next lesser power of 10.

## Results

### Homologous proteins exhibit similar thermodynamic characteristics

**Figure 1:**
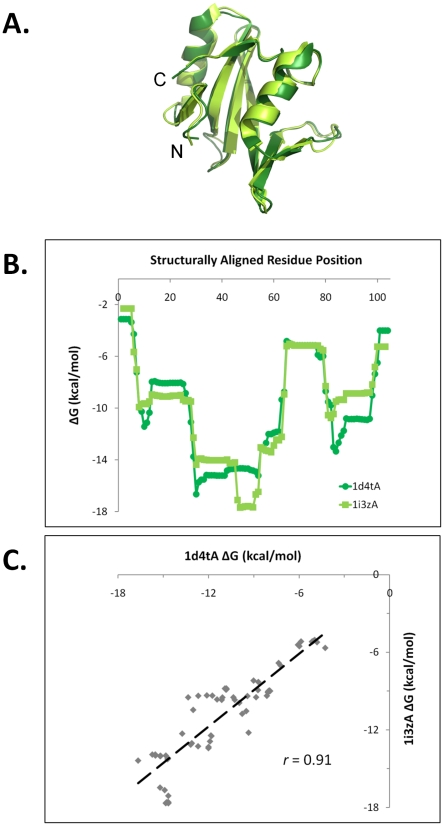
Quantitative comparison of position-specific thermodynamic stabilities between two remotely homologous proteins using pairwise structure alignment as a guide. A. Structural alignment of two SH2 domains, *Homo sapiens* Xlp protein Sap (ASTRAL domain d1d4ta [64]) and *Mus musculus* Eat2 (d1i3za [65]). The quality of the superposition is 0.95 Å RMSD over 102 CA atoms, Dali Z-score of 19.4, sequence identity of 38%; the high similarity of the two structures is evident. B. Profile comparison of local stability values (ΔG, Equation (3) as computed by the COREX/BEST algorithm), aligned according to the equivalenced residue positions from structure superposition. The high similarity of the two sets of local stabilities is evident. C. Quantitative comparison of the two sets of equivalenced local stabilities results in a Pearson correlation coefficient of *r* = 0.91. As described in Materials and Methods, the estimated probability of obtaining such a result is *p*<0.01 against Null Model 1 and p<10^−4^ against Null Model 2.

Figure 1 illustrates the methods used to compare position-specific thermodynamic descriptors of homologous (and non-homologous) protein pairs. A structural superposition of two homologous SH2-family domains, human Xlp protein SAP and mouse Eat2, is displayed in Figure 1A. The equivalenced residue pairings from this structure superposition were employed in Figure 1B to align the thermodynamic descriptors (*e.g.* local stability, ΔG) of the two proteins. A Pearson correlation of the aligned thermodynamic descriptors (Figure 1C) quantified the similarity between the two sets of descriptors. Analogous correlations were performed using the enthalpic (ΔH) and entropic (TΔS) values (data not shown). This process was repeated for all non-redundant pairwise comparisons in the structure and sequence diverse protein set, as described in Materials and Methods.

**Figure 2:**
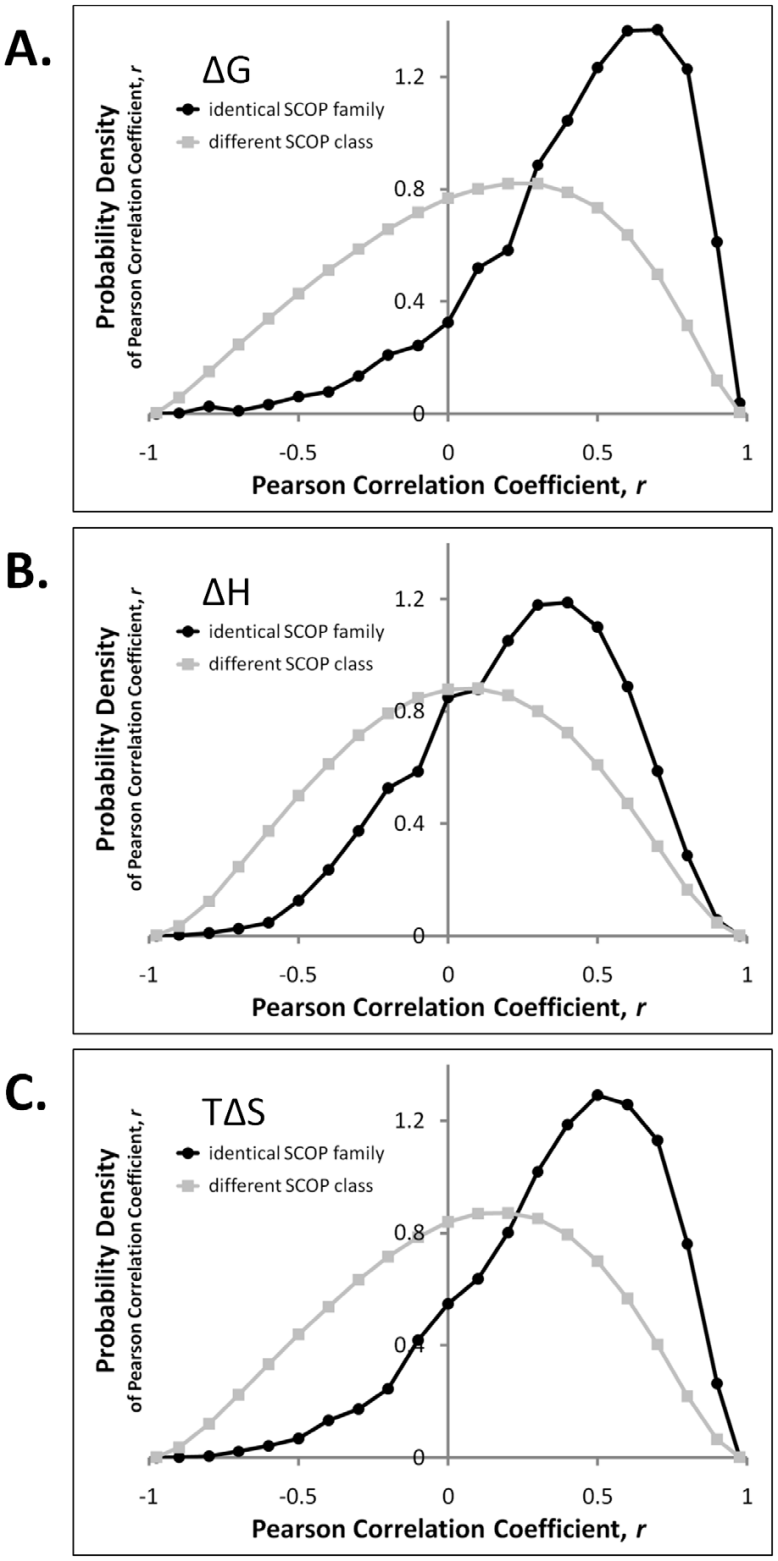
Probability densities of all *vs.* all Pearson correlations of thermodynamic descriptors between homologous and non-homologous proteins. A. Similarities in local stability (ΔG) between homologous pairs are greater than similarities between non-homologous pairs (modes of *r* = 0.61 and *r* = 0.29, respectively). B. Similarities in local enthalpy (ΔH) are also greater between homologs as compared to non-homologs (modes of *r* = 0.39 and *r* = 0.06, respectively), but the degree of similarity is less than that observed for stability. C. Similarities in local entropy exhibit a similar trend as observed for enthalpy (modes of *r* = 0.50 for homologs and *r* = 0.19 for non-homologs, respectively). Taken together, these distributions suggest that stability, enthalpy, and entropy are all conserved between homologs, but that stability is conserved to a greater extent than is enthalpy or entropy. The probability of the homolog and non-homolog distributions in each panel A – C arising from an identical underlying distribution is *p*<10^−6^, as assessed by the chi-square test for unequal numbers of points with 20 *d.o.f.* Thus, differences between homolog and non-homolog distributions are highly significant.

Because every protein in the set held a known position in the SCOP hierarchy, many comparisons could be sub-classified into either homologous (identical SCOP family) or likely non-homologous (different SCOP class) relationships. A clear pattern emerged when the correlations were tabulated for these two subsets: regardless of the thermodynamic descriptor used (*i.e.*, ΔG, ΔH, TΔS), homologous proteins exhibited significantly more highly correlated descriptors than did non-homologous proteins (Figure 2). The general absence of sequence similarity between protein pairs suggested the importance of the structural context of the position (as opposed to the identity of the amino acid at that position) in determining the energetics at each position. In quantitative terms, the mode of the homologous pairs' distribution of stability correlations was 0.61, as compared to 0.29 for the non-homologous pairs (Figure 2A and Table 1). Similarly, the modes for the enthalpy correlation distributions were 0.39 and 0.06 for homologs and non-homologs, respectively (Figure 2B). Modes for the entropy distributions were 0.50 and 0.19 for homologs and non-homologs, respectively (Figure 2C).

**Table 1:** Similarity of pairwise protein sequence, structure, and thermodynamic comparisons for each level of the SCOP hierarchy.

Level of SCOP hierarchy	*r*_ΔG_	*r*_ΔH_	*r*_TΔS_	Sequence Identity	Alignment Length	RMSD	Number of Protein Pairs
Identical SCOP family	0.61^a^	0.39	0.50	14%	66 residues	2.2 Å	8,287
Identical SCOP superfamily	0.57	0.14	0.36	11%	60 residues	2.7 Å	18,207
Identical SCOP fold	0.43	0.05	0.27	8%	60 residues	3.0 Å	54,651
Identical SCOP class	0.26	0.10	0.20	6%	30 residues	3.4 Å	1,285,025
Different SCOP class	0.29	0.06	0.19	6%	29 residues	3.6 Å	3,072,150

*^a^*All values are estimated modes of the particular probability density distribution indicated in the column heading, as described in Materials and Methods.

### Position-specific stability is correlated to a greater extent than is enthalpy or entropy

**Figure 3:**
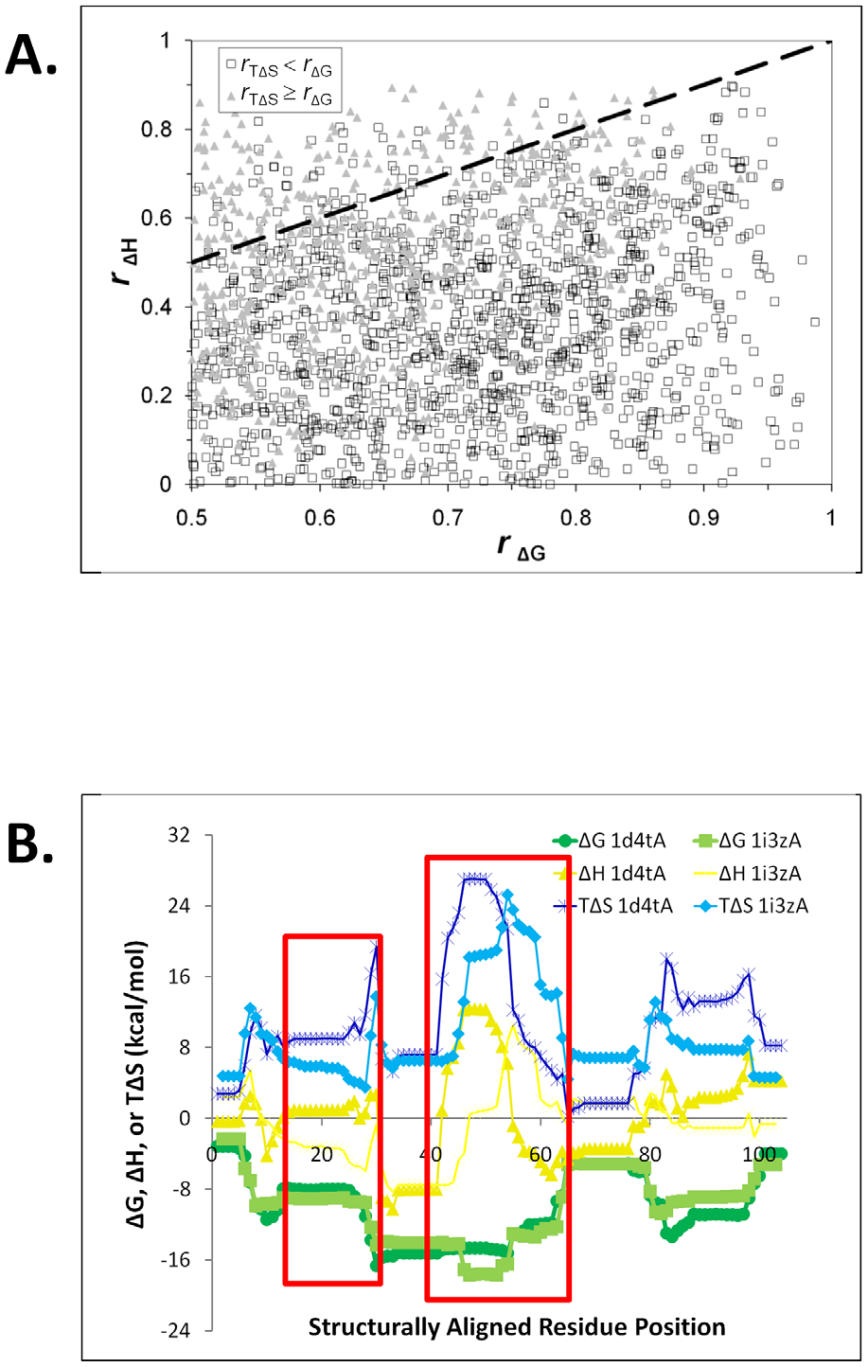
Local stability is conserved in homologous proteins to a greater extent than is component enthalpy or entropy. A. Correlation coefficients for both enthalpy and entropy are generally smaller in magnitude as compared to those for stability in the same homologous protein pair. Each point in the scatterplot represents a pair of Pearson correlation coefficients for the thermodynamic descriptors of a particular protein pair. The vast majority of enthalpy (y-axis) and entropy (triangles) points lie below an identity line (dashed) representing a given stability correlation (x-axis), demonstrating that stability is more similar than enthalpy or entropy for almost every homologous pair. B. Enthalpy and entropy of the SH2 domains shown in Figure 1 clearly demonstrate regions of large correlated changes spanning approximately 10–20 residues (boxed) in ΔH (yellow) and TΔS (blue) that result in minimal changes to ΔG (green).

Closer inspection of the correlation distributions suggested a second pattern: within homologous proteins, enthalpy and entropy generally did not exhibit correlations as great as those for stability (modes of 0.39, 0.50, and 0.61 respectively, Table 1; differences between these homolog distributions were all highly significant, exhibiting *p*<10^−6^ as assessed by chi-square tests with 19 *d.o.f*). This trend was more fully revealed by plotting individual enthalpy and entropy correlations as a function of the stability correlation for the same homologous protein pair (Figure 3A).

Examination of selected thermodynamic descriptor alignments demonstrated that the source of the differences in correlation coefficients was due to greater variation in position-specific enthalpy and entropy values as compared to the variation in stability values (Figure 3B). In particular, continuous regions of approximately 10 – 20 residues appeared to encompass much of the variation (Figure 3B, boxes). Within these variable regions, changes in enthalpy between the two proteins appeared to be somewhat balanced by changes in entropy such that the overall difference in stability was minimized (Figure 3B, boxes, discussed in detail below).

### A structural - thermodynamic “gradient” is a major organizing principle of protein homology

A clear “gradient” was observed relating structural similarity to thermodynamic correlation: as structural similarity and likelihood of homology decreased, thermodynamic similarity also decreased (Table 1). In other words, proteins of similar structure exhibited similar thermodynamic stability. Such an overall gradient was not surprising, given that it would be expected that in the limit of two identical structures, two identical COREX/BEST ensembles, and thus identical thermodynamics, would result.

However, the correlation distributions of Figure 2 showed a non-negligible degree of overlap between homologs and non-homologs. For example, approximately 10 percent of non-homologous pairs exhibited stability correlation coefficients larger than the homolog mode of 0.61, and the same percentage of homologous pairs even exhibited zero or negative correlation. There are at least two explanations for the significant overlap between the distribution of correlations for homologous and non-homologous proteins. The first is that the overlap is real and reflects actual differences between structural and thermodynamic representations of proteins. The second is that the cases of high correlation between non-homologs are a statistical artifact stemming from an enrichment of poorly described data in certain sequence stretches. To address this issue, we adopted a two-fold strategy designed to probe both for biases in the thermodynamics of the different positions associated with the correlations, as well as biases in the amino acid compositions in those positions.

First, in an effort to ensure that the overlap regions were not enriched with residue positions that occupied a particular region of thermodynamic parameter space, we performed principal components analysis (PCA) on the thermodynamic parameter space of the sequence segments that had the highest frequency of occurrence (top 10%) in the overlap regions and compared the eigenvalues to those obtained for the overall dataset, as well as for the datasets corresponding to the regions of no overlap[9]. The results (Text S1, Figure S2) revealed no bias in the overlap region, indicating that the high correlations were not driven by sequences enriched in a certain type of energetic environment.

To further investigate possible sampling bias as a source of the overlap in the distributions, we investigated the thermodynamic information content of those sequence segments that most frequently aligned with non-homologous proteins. Previously, propensities of amino acids in different thermodynamic environments were used as the basis for a fold recognition algorithm, demonstrating that the thermodynamic architecture outlined in this study represented a general framework within which to understand protein organization [10],[24],[29]. Among several noteworthy results from those studies was the ability to match all helical (or all beta) sequences to their folds (as described by a thermodynamic signature) using propensity information derived exclusively from all beta (or all helical) proteins[29], a result that demonstrated the universality of the thermodynamic representation of proteins as well as its independence from structural descriptors.

To ensure that frequently paired non-homologous sequences (*i.e.* those sequence stretches that most frequently paired with non-homologs) contained the same thermodynamic information as the overall set, we performed fold recognition experiments using thermodynamic propensities derived exclusively from those sequences. The comparable fold recognition success (Text S1, Figure S3) clearly demonstrated that the thermodynamic information content was identical across the distribution of sequences. In short, the similarity in both the range of thermodynamic parameter space occupied, as well as the distribution of amino acids within this parameter space between sequences that frequently correlate with non-homologs and those that do not, suggested that the overlap regions in the distributions shown in Figure 2 are not statistical artifacts. Instead, the results may provide insight into the relationship between structure, energy, and the evolution of this diverse library of folds. This point is discussed in more detail below.

### Exceptions to shared structure and thermodynamic similarity between homologous proteins

As expected, inspection of the proteins contained in the overlap regions in Figure 2 revealed interesting exceptions to the overall structural-thermodynamic gradient, exceptions that required a more nuanced interpretation of the gradient. More generally, these exceptions suggested an organizational framework for the integration of thermodynamic information into existing fold classification schemes (as described below). The exceptions could be broadly ordered into at least three distinct classes: 1) non-homologous proteins that contained regions of coincident structural and thermodynamic similarity, 2) homologous proteins containing regions of thermodynamic similarity and structural dissimilarity, and 3) homologous proteins containing regions of structural similarity and thermodynamic dissimilarity.

**Figure 4:**
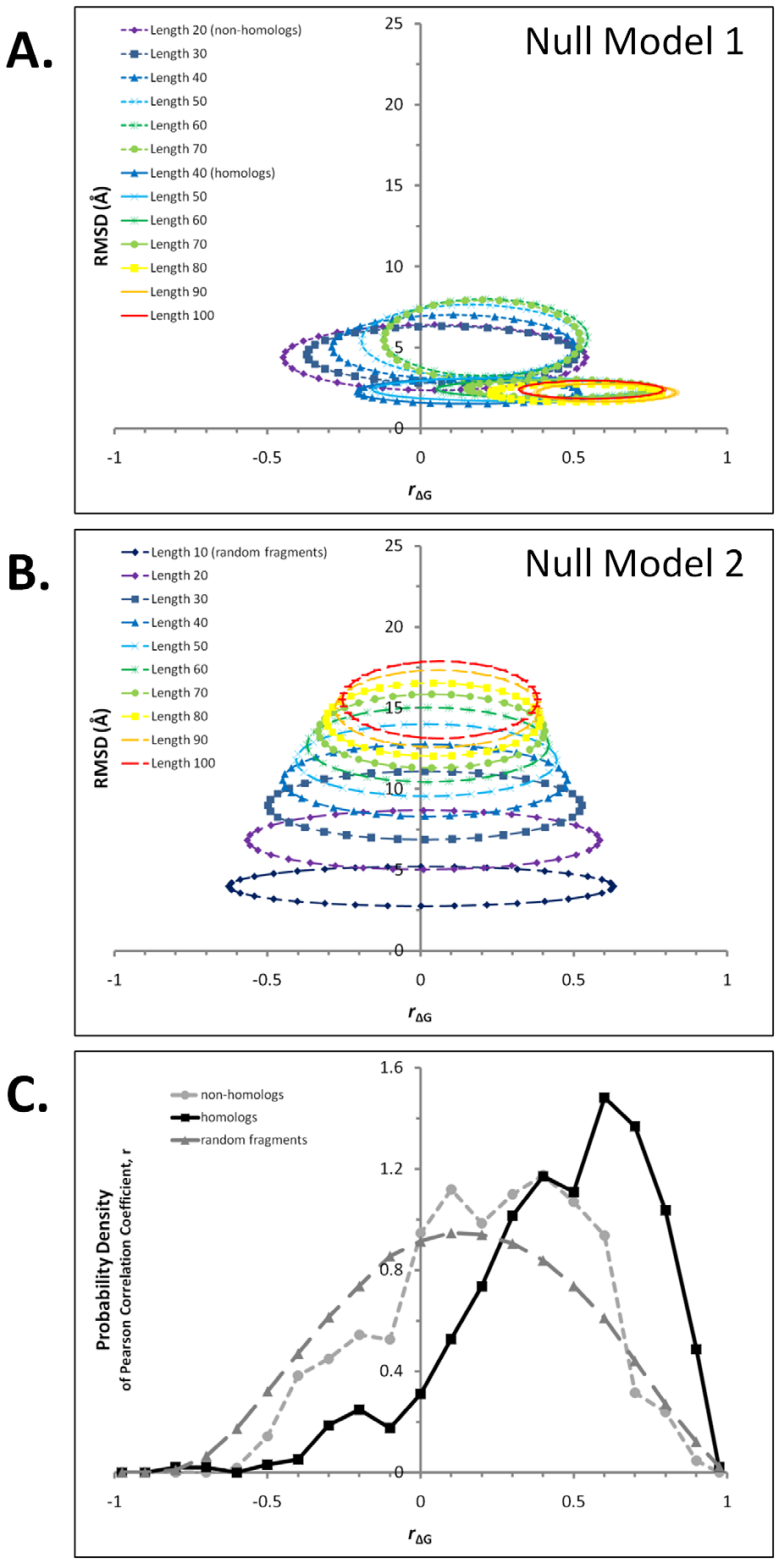
Null models to estimate probability of particular levels of thermodynamic similarity in the presence or absence of structural similarity. Panel A represents background distributions of homology-specific subsets of Null Model 1, and panel B represents the background distribution of Null Model 2. In each of these panels, ellipses centered on the mean values of structural and thermodynamic similarity for a given alignment length are displayed. The semi-axes of a particular ellipse each correspond to one standard deviation of the similarity values for that length set. Comparison of each panel conveys a visual sense of the structural/thermodynamic probability space covered by each null model: Null Model 2 covers substantially more probability space than does Null Model 1. A. Background distributions for homologous protein pairs are displayed as solid lines, background distributions for non-homologous pairs as dotted lines. Structural alpha-carbon RMSD is almost independent of homolog alignment length, while similarity in local thermodynamic stability increases with length. In contrast, RMSD, as well as thermodynamic similarity, decreases with alignment length for non-homologs. B. Background distribution for randomly drawn fragments. RMSD increases with alignment length while thermodynamic similarity decreases. At lengths less than approximately 20 residues, a substantial probability for either correlated or anti-correlated thermodynamic stability exists. C. Projections of null model distributions into the thermodynamic stability dimension. Probability density functions from Null Model 1 for homologs (solid line) and non-homologs (dotted line) are displayed, as well as from Null Model 2 for randomly drawn fragments (dashed line). Each of these distributions is composed of alignments of lengths 70–75 residues. Progressive rightward shifting of the non-homologous and homologous distributions relative to random suggests the importance of structural context to thermodynamic similarity, as discussed in the text.

To facilitate quantitative description of these exceptional cases, two empirical probability models of thermodynamic similarity were constructed to assess how often these cases might be expected due to chance, as described in Materials and Methods and displayed in Figure 4. These models could be regarded as occupying extremes in structural and thermodynamic similarity space and consequently resulted in different probability estimates. The first model (Null Model 1) accounted for the interdependence of structural and thermodynamic similarity at each alignment length. *P*-values for homologs and non-homologs were determined separately at each length by comparing the specific combination of structural and thermodynamic similarities with the frequency of obtaining such a combination across all comparisons. The density of points is summarized in Figure 4A for different alignment lengths. We note that the comparisons in Null Model 1 are DALI-aligned structures and thus represent comparisons between sequence stretches that have been selected for high structural similarity. To determine the probability of obtaining a particular thermodynamic correlation across any sequence comparison in the database, a second null model (Null Model 2) was adopted. According to Null Model 2, length-matched gapless alignments of randomly paired protein fragments were examined, a step taken to reduce the interdependence of structural and thermodynamic similarity. The Null Model 2 exhibited an inverse dependence of structural and thermodynamic similarity on length, in particular revealing that alignments of less than 20 residues had a substantial probability of high positive or negative thermodynamic correlation (Figure 4B). Because the background distribution of Null Model 2 covered a larger amount of structural/thermodynamic similarity space, *p*-values estimated from Null Model 2 were generally more significant, as compared to Null Model 1. Projections of these two-dimensional null model distributions into the single dimension of thermodynamic stability similarity, for alignments of approximately 70 residues in length, are displayed in Figure 4C.

As Figure 4C reveals, the probability density of stability correlation coefficients for random alignments of approximately 70 residue stretches (Null Model 2) is centered on zero, with approximately 80% of the comparisons falling below correlations of 0.5. As expected, the probability density functions of structurally aligned sequences for both non-homologs and homologs are shifted to higher correlations, with the shift for homologs being more dramatic. The significance of this result is discussed in more detail below. For now we simply note that these distributions can be used to identify statistically significant exceptions to homologous structural and thermodynamic similarity and to investigate the possible biological and evolutionary relevance of such examples.

**Figure 5:**
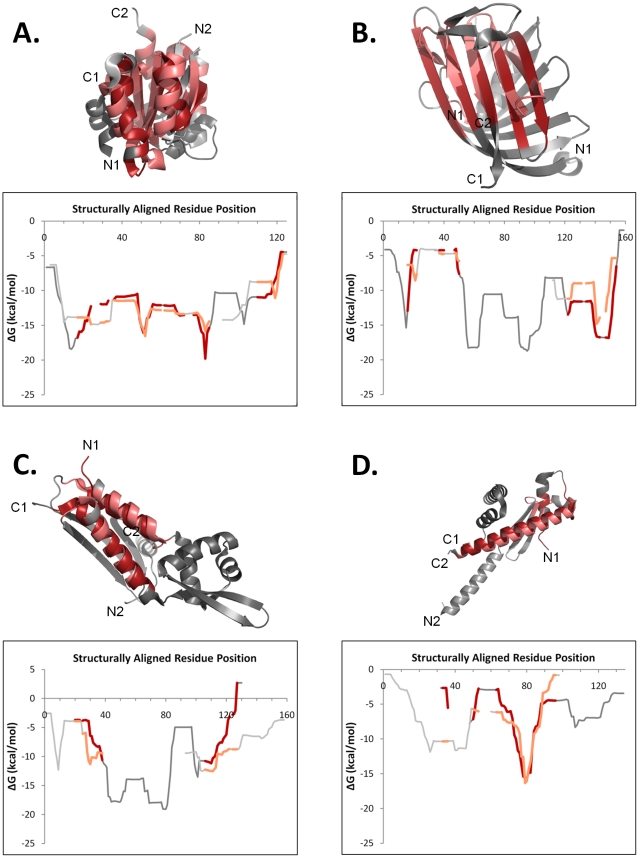
Four examples of non-homologous protein pairs that show regions of similar structure and thermodynamic stability. Examples of this type are a modest fraction (approximately 10%) of thermodynamically similar comparisons (*r*_ΔG_>0.61) between non-homologous proteins. It is not clear at present if these similarities are entirely analogous or homologous in nature. In each panel, the DALI structure superposition (top) and the aligned thermodynamic stability profile (bottom) are displayed. Structurally similar regions exhibiting similar thermodynamics are colored shades of red; unaligned regions are colored shades of gray. The first protein named in every pair is colored darker than the second protein. Chain termini are labeled. A. *Methanocaldococcus jannaschii* ribosomal protein L7ae (d1sdsa [66], bacillus chorismate mutase-like fold) and *Caulobacter crescentus* DivK (d1mb3a [67], flavodoxin-like fold); two β-α-β units (RMSD of 5.0 Å over 70 aligned residues) within dissimilar overall structures share similar local stabilities (*r_ΔG_* = 0.77, Null Model 1 *p*<0.20, Null Model 2 *p*<10^−4^). B. *Bacillus subtilis* YwiB (d1r0ua (unpublished), lipocalins fold) and *Serratia marcescens* chitinase insertion domain (d1edqa3 [68], FKBP-like fold); one β-hairpin and one three-stranded β-sheet form a larger sheet (RMSD of 3.9 Å over 35 aligned residues) with similar local stabilities (*r_ΔG_* = 0.85, Null Model 1 *p*<0.10, Null Model 2 *p*<10^−3^) in the context of dissimilar overall structures. C. *Archaeoglobus fulgidus* AF2008 (d1sfxa (unpublished), DNA/RNA binding three helical bundle) and *Saccharomyces cerevisiae* YBL001C (d1lxja [69], ferredoxin-like fold); two antiparallel α-helices (RMSD of 3.3 Å over 34 aligned residues) exhibit similar local stabilities (*r_ΔG_* = 0.75, Null Model 1 *p*<0.06, Null Model 2 *p*<10^−4^) within dissimilar overall structures. D. *Gallus gallus* histone H2B (d1tzyb [70], histone fold) and *H. influenzae* HI0442 (d1j8ba [71], YbaB-like fold); one long amphipathic α-helix (RMSD of 4.5 Å over 37 aligned residues) has similar local stabilities (*r_ΔG_* = 0.87, Null Model 1 *p*<0.03, Null Model 2 *p*<10^−4^) in the context of two different folds.

Several examples of non-homologous proteins that nonetheless exhibited correlated position-specific stability are displayed in Figure 5. These examples were representative of approximately 10% of non-homologs with high thermodynamic correlation (defined as those above the homolog mode stability correlation value of 0.61, about 10% of the total non-homologs), in that they contained structurally and thermodynamically similar regions within otherwise dissimilar proteins. Some specific types repeatedly observed were β-α-β units (Figure 5A), non-local β-hairpins forming a sheet (Figure 5B), antiparallel helices (Figure 5C), and amphipathic single helices (Figure 5D).

**Figure 6:**
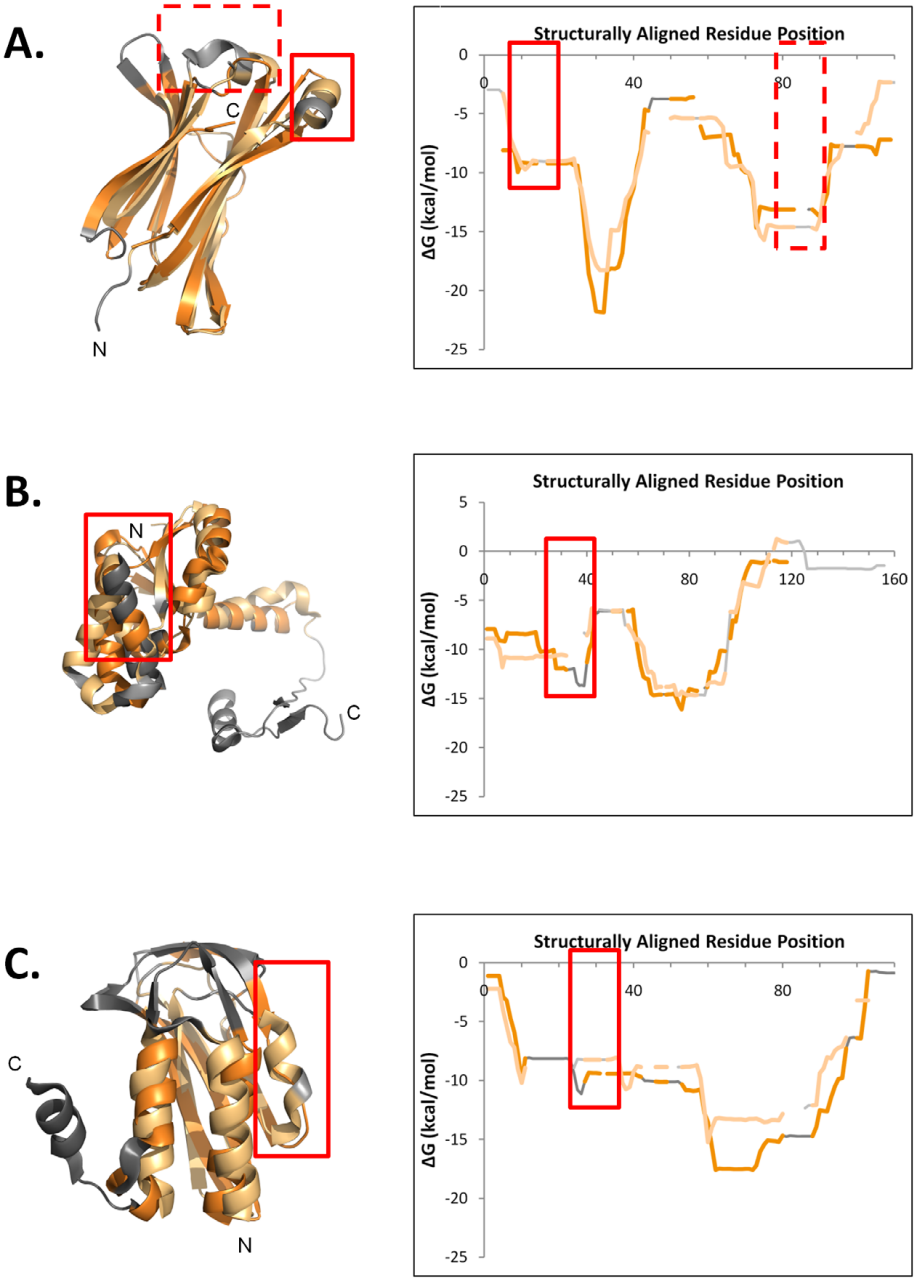
Three examples of remotely homologous proteins exhibiting regions of secondary structure change with concomitant minimal disruption of the local stability. These examples illustrate a plausible thermodynamically-mediated process of evolutionary fold change. Interestingly, the boxed regions sometimes, though not always, have functional associations, possibly related to the structural change, as described in the text. In each panel, the DALI structure superposition (top) and the aligned thermodynamic stability profile (right) are displayed. Structurally similar regions exhibiting similar thermodynamics are colored shades of orange; unaligned regions are colored shades of gray. The first protein named in every pair is colored darker than the second protein. Chain termini are labeled. A. Immunoglobulin C1 set domains, murine cytomegalovirus m144 protein, alpha 3 domain (d1pqza1, (Miley, M. J. and Fremont, D. H., unpublished)) and *H. sapiens* CL-lambda domain (d1rzfl2, [72]). The solid boxed region, located near the N-terminal end of the proteins, has functional significance for m144 binding to β-2 microglobulin, [37], but its function, if any, in the human protein is not known. The RMSD of the structural alignment is 2.6 Å over 82 residues, the *r_ΔG_* = 0.92 (Null Model 1 *p*<0.02, Null Model 2 *p*<10^−5^). B. Aspartate/glutamate racemase family, *Pyrococcus horikoshii* aspartate racemase (d1jfla2, [38]) and *Aquifex pyrophilus* glutamate racemase (d1b74a2, [39]). The boxed region encompasses the dimerization interface for both proteins, but each protein is known to have a different dimerization mode [38], possibly related to the structural change. The RMSD of the structural alignment is 3.7 Å over 89 residues, the *r_ΔG_* = 0.92 (Null Model 1 *p*<0.03, Null Model 2 *p*<10^−5^). C. *E. coli* biotin carboxylase C- domain (d1dv1a1, [73]) and *E. coli* N5-carboxyaminoimidazole ribonucleotide synthetase PurK (AIRC) C-domain (d1b6ra1, [74]). Although the boxed region also encompasses part of the dimerization interfaces for these two molecules, it is not known whether the structural differences are of functional significance. The RMSD of the structural alignment is 2.8 Å over 64 residues, the *r_ΔG_* = 0.90 (Null Model 1 *p*<0.03, Null Model 2 *p*<10^−5^).

**Figure 7:**
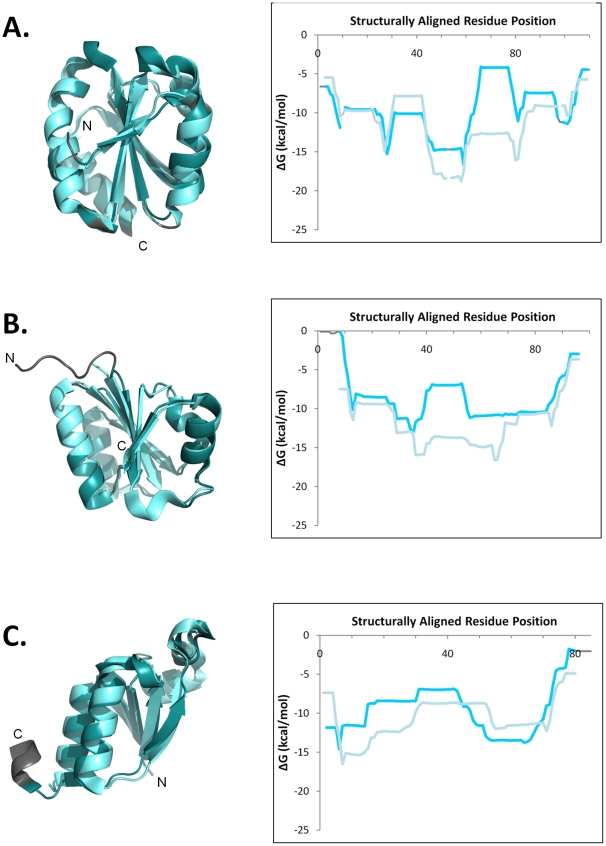
Three examples of remotely homologous proteins that are extremely structurally similar, yet the local stabilities exhibit substantial differences. This class of thermodynamic relationships also may have functional importance, as described in the text. In each panel, the DALI structure superposition (left) and the aligned thermodynamic stability profile (right) are displayed. Structurally similar regions exhibiting similar thermodynamics are colored shades of cyan; unaligned regions are colored shades of gray. The first protein named in every pair is colored darker than the second protein. Chain termini are labeled. A. *E. coli* thioredoxin (d2trxa, [75]) and *H. sapiens* thioredoxin (d1aiua, [41]); the region of greatest stability difference contains the functionally important conserved Cys73 in the human protein, absent in the bacterial protein. The RMSD of the structural alignment is 1.2 Å over 95 residues, the *r_ΔG_* = 0.45 (Null Model 1 *p*<0.04, Null Model 2 *p*<10^−5^). B. *H. influenzae* (d1p3da1, [76]) and *T. maritima* (d1j6ua1, [77]) MurCD N-terminal domains; the greater stability of the thermophilic protein is evident. The RMSD of the structural alignment is 1.0 Å over 81 residues, the *r_ΔG_* = 0.41 (Null Model 1 *p*<0.03, Null Model 2 *p*<10^−5^). C. *Anopheles dirus* b class delta GST N-terminal domain (d1jlva2, [78])and *Arabidopsis thaliana* class zeta GST N-terminal domain (d1e6ba2, [42]); the N-terminal region of greatest stability difference contains a novel active site and conserved residues (Ser17 and Cys19) in the zeta class protein, resulting in a novel function [42]. The RMSD of the structural alignment is 1.2 Å over 71 residues, the *r_ΔG_* = 0.51 (Null Model 1 *p*<0.03, Null Model 2 *p*<10^−5^).

Additional statistically significant exceptions to the structural-thermodynamic gradient, involving homologous proteins, are displayed in Figures 6 and 7. Figure 6 shows three instances of homologous pairs exhibiting conserved local stability despite secondary structure variation. This phenomenon has been previously identified as a possible thermodynamic mechanism for evolutionary fold change[9], and the examples seen here, occurring in a variety of secondary structural contexts, suggest its generality.

However, a novel hypothesis is that these regions of thermodynamically conserved structure change possibly coincide with regions of functional importance; this hypothesis is illustrated with several examples. Figure 6A shows the structure superposition and aligned stability profiles of two immunoglobulin C1-set domains. Highlighted are two boxed regions where stability is conserved despite sequence and structure variation; one region contains functional residues involved in binding of the murine cytomegalovirus m144 protein, alpha 3 domain to the β2m subunit [37]. Figure 6B highlights a strand to helix conversion between aspartate and glutamate racemases, located in a region known to mediate the different dimerization modes of the two enzymes [38],[39]. Similarly, Figure 6C highlights a region of structure change important for dimerization in each of two biotin carboxylase C-terminal domain-like proteins.

In contrast, Figure 7 shows three statistically significant examples of homologous protein pairs whose native state structures were quite similar (RMSD ≈1 Å) and yet exhibited low or modest thermodynamic stability correlations (*r_ΔG_*≤0.5). One similar example of thermodynamic dissimilarity in the context of high structural similarity has recently been experimentally confirmed using point mutations of *Escherichia coli* adenylate kinase[40]. As suggested by the relatively small area of negative correlations between homologs in Figures 2A–C, structure similarity in the absence of thermodynamic similarity did not occur very often between homologous proteins in the database (only 8% of homologous pairs with an RMSD <2.5 Å exhibited a negative correlation coefficient).

Despite its relatively low frequency of occurrence, this class of exceptions to the structural-thermodynamic gradient also may have functional relevance, as illustrated by several examples. Displayed in Figure 7A are the superposition and aligned stability profiles of two extremely structurally similar thioredoxins from *E. coli* and human, with an RMSD of 1.2 Å over 122 CA atoms. However, the stability profiles are only weakly correlated (*r* = 0.45), largely due to stability differences in the middle half of the proteins' alignment. The region of largest difference (approximately alignment positions 60 – 80) encompasses the conserved Cys 73 residue, not found in the *E. coli* protein, which facilitates a unique and functionally important dimer form of human thioredoxin [41]. Figure 7B shows the comparison between two MurCD N-terminal domains from *Haemophilus influenzae* and the thermophile *Thermotoga maritima*; the low correlation between stability profiles clearly results from the greater predicted stability of the thermophile. Similarly, the stabilized N-terminal region of the zeta-class GST N-terminal domain shown in Figure 7C reduces the correlation with its delta-class homolog's stability profile. The predicted increase in stability is possibly related to the region's unique active site residues and associated novel functionality noted for the zeta-class [42].

## Discussion

Position-specific thermodynamic attributes of proteins, such as local stability, enthalpy, and entropy, are preserved to a large degree in remote (*i.e.*, twilight-zone sequence identity and below) homologs. One implication of this result is that thermodynamics reinforces structure and sequence similarity, suggesting that thermodynamic attributes are likewise evolutionarily conserved properties. Upon closer inspection, however, several important features of the current analysis emerge regarding the relationship between the conservation of structure and energy. As noted above, Figure 4C reveals the shifting of the probability density function for non-homologs and homologs when comparisons are made with DALI-aligned structures, relative to random alignments. The shift observed for the non-homologs relative to the random sequence comparison is expected. In anecdotal terms, this result indicates that a particular stretch of structural elements (*e.g*., a helix-loop-helix) will have more similar energetics than two stretches of randomly selected structure. Perhaps surprisingly, the energetic correlation for homologs is improved over the non-homologs for a given sequence length (even though homologs with substantial sequence similarity were specifically not included in the analysis). This latter result is important because the difference between the improvement between homologs and nonhomologs provides a quantitative measure of the impact of the “structural context” of the specific sequences being compared. This is noteworthy because it undermines the notion that thermodynamic identity is defined by the RMSD of the structural units being compared. To the contrary, the results suggest a great deal of energetic heterogeneity for a particular structural motif. In other words, not all helix-loop-helix motifs of a given length and structural similarity, for example, will be thermodynamically equal. In fact, over the entire database, the results not only reveal significant instances of energetic heterogeneity for a specific structural motif, but more importantly, energetic similarity between different structures. It is our hypothesis, which we are currently testing, that it is precisely this context dependence of the energies of structural elements that determines how different folds can evolve from parental folds and why minimal sequence changes can dramatically change a protein fold[43]–[45].

Another implication of the conservation of local stability in remotely homologous proteins suggests that some aspect of protein behavior vital to the robustness of the organism is contingent on maintaining the regional stability. There are at least two possible reasons for such conservation. First, it is possible that a specific balance of regional stability within a protein may bias (or preclude) certain folding pathways, thus rendering the stability hierarchy in the protein critical to maintaining folding fidelity [46],[47]. Second, and perhaps more prevalent, is that the locally unfolded state plays an important functional role. Indeed, locally unfolded states have been shown to be functionally important in numerous native state ensembles, mediating catalysis [48],[49], allostery [50],[51], and signaling transduction [22],[52].

Intriguingly, exceptions to the trend of thermodynamic conservation exist, just as they are already known to exist for structure or sequence (*i.e.* homologous sequences are able to adopt unrecognizably different structures [44],[53] and homologous structures can result from unrecognizably different sequences [54],[55], respectively). As suggested by the examples given in Figures 6 and 7, these exceptions to thermodynamic conservation may be evolutionarily or functionally important, despite their low frequency of occurrence.

One interesting type of exception found here is that position-specific enthalpy and entropy are less conserved than stability. This observation suggests that in regions where this phenomenon occurs, the overall stability is more important than the thermodynamic mechanism utilized to achieve that stability. It is tempting to speculate that amino acid mutation driven changes in local entropy and enthalpy balance in conservation of local stability, as seen in Figure 3B. However, such “enthalpy-entropy compensation”, long reported in proteins as well as other chemical systems, has a controversial history [56]–[59], with the apparent compensation being due (in many cases) to errors in enthalpy and entropy that are effectively amplified when the free energy is determined from the difference between them. Thus, although it is possible that such balance is somehow a mathematical artifact [60], there is currently no evidence for such an artifact in the current analysis.

Other types of exceptions, in the form of stability differences, may arise from differences of structure, organism temperature, or functionality. It is also possible that thermodynamic similarities between putative non-homologs, now treated as “exceptions” (*i.e.* thermodynamic analogy), may reveal heretofore unknown evolutionary relationships.

We propose in this regard that thermodynamics can mediate mechanisms for evolutionary fold change[9]. In other words, local native state structure change between two homologous proteins is possible without major disruption of local stability and, possibly, enthalpy or entropy. Conversely, functional or temperature adaptation can be achieved by changing the thermodynamics of excited state conformational fluctuations without disrupting the ground (native) state structure[40]. These two complementary processes may be thought of as ways to affect function by “sculpting” (*i.e.*, changing the size, shape, and energetic properties of) a protein's native state ensemble. Future experimental work will be directed at ways to intelligently employ these processes in protein design and engineering.

Finally, we note that considerable debate has emerged regarding whether protein fold space is continuous or discontinuous [61]–[63], with a major limiting factor in its resolution being the absence of a metric that can quantitatively compare different structures within a unified framework. One potential benefit of the unique thermodynamic representation of protein fold space used here is that it provides a quantitative connection between protein stability and fold specificity, in effect providing a vehicle for directly addressing this question. Indeed, this discovery of conserved position-specific thermodynamics not only furthers our understanding of the role of energetics in protein structure, function, and evolution, but also suggests an organizational framework for a possible thermodynamically-informed classification of protein homology.

## Supporting Information

Text S1 Supporting Information Text
(0.05 MB DOC)

Figure S1 Contributions of solvent and conformational terms to the total native state entropy. Profile comparison of local total entropy, solvation entropy, or conformational entropy values for two SH2 domains, human Xlp protein Sap (d1d4ta) and mouse Eat2 (d1i3za). Entropies were computed by the COREX/BEST algorithm, using Equation (5) from the main text. The SH2 domains are the same as those displayed in Figures 1 and 3 of the main text, aligned according to the equivalenced residue positions from the DALI structure superposition as described in the corresponding figure legends. Similarity of the various local entropies, as well as the dominant contribution of solvation entropy to the total, is evident.
(1.35 MB TIF)

Figure S2 Eigenvectors and percent variance of thermodynamic descriptors space for subsets of residue positions. Subsets of residue positions were constructed from regions of overlap, as described in the text. The percent variance explained by each eigenvector is shown in parentheses. All eigenvectors and percent variances are similar between each subset of residue positions and the full data set. A. Values for eigenvector 1. B. Values for eigenvector 2.
(0.94 MB TIF)

Figure S3 Thermodynamic fold recognition results for fifty randomly chosen query proteins using information derived from subsets of residue positions. Subsets of residue positions were constructed from regions of overlap, as described in the text. A. Distributions of percentile rank for fold-recognition Z-scores between a thermodynamically defined query and its correct amino acid sequence. Successful fold recognition experiments exhibit lower percentile ranks. Distributions of percentile ranks are similar regardless of the subset source of thermodynamic information. B. Correlation of fold recognition raw scores between experiments using different subset sources of thermodynamic information. Scores are similar (visibly correlated) regardless of the subset source of thermodynamic information.
(1.04 MB TIF)
