# Supplementary material for: Investigating Homology between Proteins using Energetic Profiles
Source: PLoS Comput Biol. 2010 Mar 26;6(3):e1000722. doi: 10.1371/journal.pcbi.1000722 (PMC2845653; doi:10.1371/journal.pcbi.1000722)
Supplement: Text S1 — Supporting Information Text (0.05 MB DOC) [file pcbi.1000722.s001.doc]

**Supporting Information for the manuscript “Investigating homology between proteins using energetic profiles” by James O. Wrabl and Vincent J. Hilser.**

Contributions of solvent and conformational terms to the total native state entropy

Equation (5) of the main text demonstrates that the total entropy of the COREX / BEST statistical thermodynamic treatment is composed of both solvent and conformational entropy terms. Under the native state conditions simulated in this work, these terms make opposing, but unequal, contributions to the total entropy. For the two proteins displayed in Figure 1 of the main text, the local solvation entropy at any particular residue position is approximately 1.5 times larger than the conformational entropy (Figure S1). In addition, the solvation entropy varies to a greater extent than does the conformational entropy, resulting in a total entropy that is dominated by the solvation component.

Investigation of possible statistical bias in the thermodynamics of overlap regions

We investigated the possible presence of bias in the position-specific thermodynamic descriptors (ΔG, ΔH, and TΔS) in populations of residue positions frequently occurring in the overlap regions of Figure 2 of the main text. Two approaches were employed. The first approach used principal components analysis (PCA)[1,2] to mathematically describe the shape of the three-dimensional thermodynamic space of overlap regions. Comparison with the descriptions for non-overlap regions could reveal differences in the thermodynamic properties of each region, thus implying that the observed differences between thermodynamics of homologs and non-homologs resulted from statistical bias and thus may not be biologically relevant. The second approach used thermodynamic fold recognition experiments[3,4] where the information used to match sequence to structure was derived from either overlapping or non-overlapping regions. Differences in the fold recognition effectiveness could likewise suggest that the thermodynamic information in each region was biased and therefore also not biologically relevant.

Principal components analysis of local thermodynamic descriptors from residue positions frequently occurring in overlap regions

PCA was performed on the entire set of thermodynamic data from 3,866 proteins, and on four subsets of data representing residue positions enriched in one of four overlap categories: 1) homologs that were similar in position-specific stability (ΔG), 2) homologs that were dissimilar in local stability, 3) non-homologs that were similar in local stability, and 4) non-homologs that were dissimilar in local stability. These four categories were defined according to the mode of the stability correlation coefficient for homologous pairs reported in Table 1 of the main text, *r_ΔG_* = 0.61. The four overlap categories were constructed as follows. The two sets (Table 1 of the main text) of 8,287 homologous pairs aligned by DALI and 3,072,150 non-homologous pairs were each subdivided depending on if the stability correlation coefficient of the pair was greater or less than *r_ΔG_* = 0.61. Then, for each DALI-aligned position in every protein of each subset, the number of times the position aligned to any other position in the subset was counted. In this way, residue positions could be ranked within each subset according to how frequently they participated in structural and thermodynamic alignments characteristic of the subset. The most frequent 10 % of residue positions within each subset, representing populations possibly most enriched in thermodynamic bias were considered for PCA analysis. The final sizes of each 10 % subset, as compared to the complete set of 362,516 residue positions, were 1) 7,921 positions enriched in homologs of similar position-specific stability, 2) 9,212 positions enriched in homologs of dissimilar stability, 24,156 positions enriched in non-homologs of similar stability, and 4) 21,654 positions enriched in non-homologs of dissimilar stability. No more than 40 % of the residue positions were identical between any of the four subsets. The eigenvectors and eigenvalues computed from the entire set and the four subsets were essentially identical (Figure S2), demonstrating that the thermodynamic information within each subset was generally similar to that of the entire set. In particular, the thermodynamic information contained within overlap regions in Figure 2 was not distinguishable from that contained within the non-overlap regions. Thus, it was unlikely that high or low thermodynamic similarity resulted from enrichment of a region with specific type of energetic environment.

Thermodynamic fold recognition experiments based on information contained in residue positions frequently occurring in overlap regions

Thermodynamic fold recognition experiments were performed as previously described.[4] Differences from the previous protocol are detailed in the following. The 362,516 residue positions of the 3,866 proteins in the complete set were clustered into eight thermodynamic environments, based on their position-specific ΔG, ΔH, and TΔS values using the Partitioning Around Medoids algorithm as implemented in the S-PLUS 6.0 software package (Insightful Corporation). Four subsets of the complete data representing residue positions enriched in one of four overlap categories were extracted: 1) homologs that were similar in position-specific stability (ΔG), 2) homologs that were dissimilar in position-specific stability, 3) non-homologs that were similar in position-specific stability, and 4) non-homologs that were dissimilar in position-specific stability. These subsets were similar in spirit to the four subsets used in Figure S2 but were constructed using a different procedure. Each DALI-aligned residue position in the two sets of 8,287 homologous pairs and 3,072,150 non-homologous pairs was ranked within each subset according to the following score:

Score = *N* x (average Pearson stability correlation coefficient *r_ΔG_*) (S1)

In Equation (S1), *N* is the number of times the residue was structurally aligned with any other residue in the set, and the term in parentheses is the average correlation coefficient over all DALI alignments in the subset containing the residue. Therefore, the positions with the highest scores exhibited both a high frequency of occurrence in DALI alignments as well as highly similar position-specific stability within these alignments. Conversely, positions with a low score were aligned infrequently with proteins concomitantly exhibiting low thermodynamic similarity. The top and bottom 10 % subsets of residue positions ranked with Equation S1 in each of the homologous and non-homologous sets were considered to be enriched in residues corresponding to the four overlap categories. The final sizes of each 10% subset, as compared to the complete set of 362,516 residue positions, were 1) 11,914 positions enriched in homologs of similar position-specific stability, 2) 11,914 positions enriched in homologs of dissimilar stability, 3) 24,156 positions enriched in non-homologs of similar stability, and 4) 24,156 positions enriched in non-homologs of dissimilar stability.

Fifty query proteins were chosen randomly from the complete 3,866 protein set. Residue positions in each protein were assigned to one of the eight thermodynamic environments defined in the clustering. Fold recognition experiments were performed using each of the fifty proteins as query against the database of 3,688 protein sequences, with the log-odds scoring matrix in each experiment derived from one of the subsets of residue positions described above. Any residue positions contained in both the query and the subset were omitted from the log-odds matrix. In every experiment, the 3,866 amino acid sequences were individually optimally aligned with the thermodynamic environments of the query, subject to the log-odds scoring matrix and the Smith-Waterman dynamic programming algorithm as implemented in the PROFILESEARCH software package.[5,6] The percentile rank of the correct sequence’s PROFILESEARCH Z-score was recorded and used as the metric of fold-recognition efficacy. As shown in Figure S3A, every subset of residue positions resulted in similar fold recognition success over the test set of 50 query proteins. Moreover, PROFILESEARCH raw scores for each query were highly correlated between subsets of residue positions and the entire set (Figure S3B). These results suggest that similar thermodynamic information was contained in the overlapping and non-overlapping regions of Figure 2.

**Supporting Information References**

1. Manly BFJ (1986) Multivariate statistical methods: a primer. New York: Chapman and Hall.

2. Vertrees J, Wrabl JO, Hilser VJ (2009) An energetic representation of protein architecture that is independent of primary and secondary structure. Biophys J 97: 1461-1470.

3. Wrabl JO, Larson SA, Hilser VJ (2002) Thermodynamic environments in proteins: fundamental determinants of fold specificity. Protein Sci 11: 1945-1957.

4. Larson SA, Hilser VJ (2004) Analysis of the "thermodynamic information content" of a Homo sapiens structural database reveals hierarchical thermodynamic organization. Protein Sci 13: 1787-1801.

5. Bowie JU, Luethy, R., Eisenberg, D. (1991) A method to identify protein sequences that fold into a known three-dimensional structure. Science 253: 164-170.

6. Gribskov M, Luethy, R., Eisenberg, D. (1990) Profile analysis. Methods in Enzymology 183: 146-159.
