# Supplementary figures and images for: Investigating Homology between Proteins using Energetic Profiles
Source: PLoS Comput Biol. 2010 Mar 26;6(3):e1000722. doi: 10.1371/journal.pcbi.1000722 (PMC2845653; doi:10.1371/journal.pcbi.1000722)

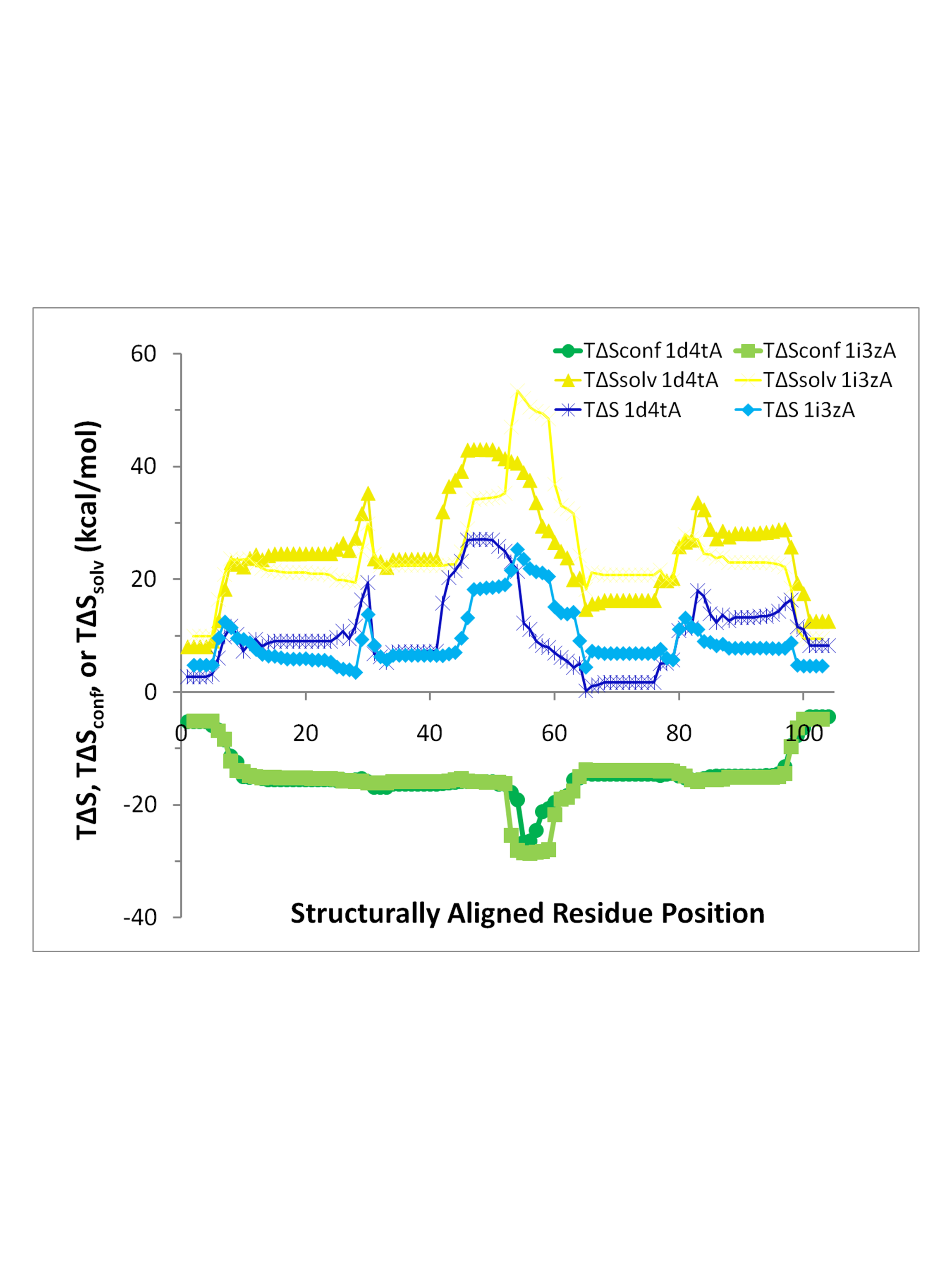

Supplement: Figure S1 — Contributions of solvent and conformational terms to the total native state entropy. Profile comparison of local total entropy, solvation entropy, or conformational entropy values for two SH2 domains, human Xlp protein Sap (d1d4ta) and mouse Eat2 (d1i3za). Entropies were computed by the COREX/BEST algorithm, using Equation (5) from the main text. The SH2 domains are the same as those displayed in Figures 1 and 3 of the main text, aligned according to the equivalenced residue positions from the DALI structure superposition as described in the corresponding figure legends. Similarity of the various local entropies, as well as the dominant contribution of solvation entropy to the total, is evident. (1.35 MB TIF) [file pcbi.1000722.s002.tif]

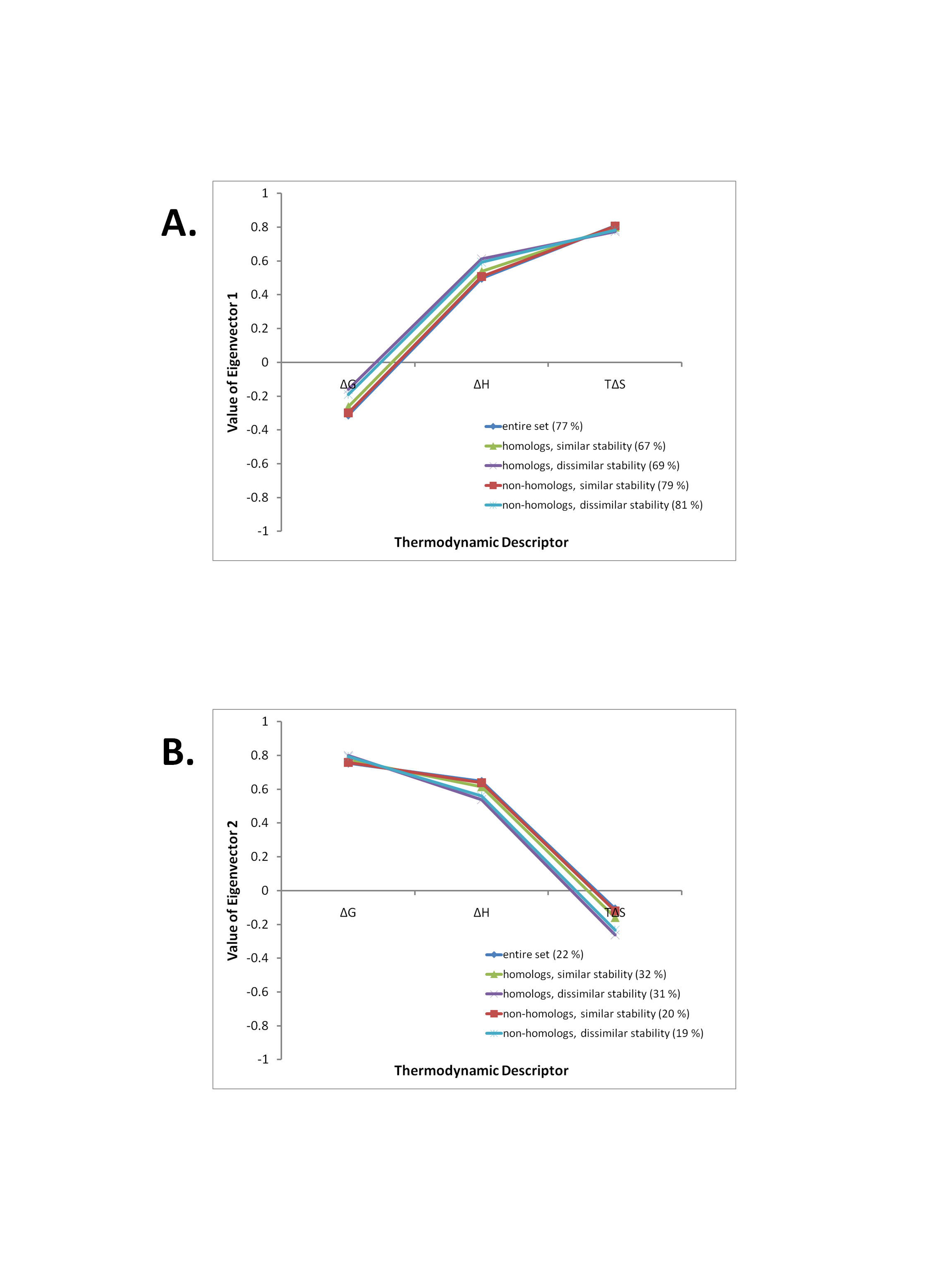

Supplement: Figure S2 — Eigenvectors and percent variance of thermodynamic descriptors space for subsets of residue positions. Subsets of residue positions were constructed from regions of overlap, as described in the text. The percent variance explained by each eigenvector is shown in parentheses. All eigenvectors and percent variances are similar between each subset of residue positions and the full data set. A. Values for eigenvector 1. B. Values for eigenvector 2. (0.94 MB TIF) [file pcbi.1000722.s003.tif]

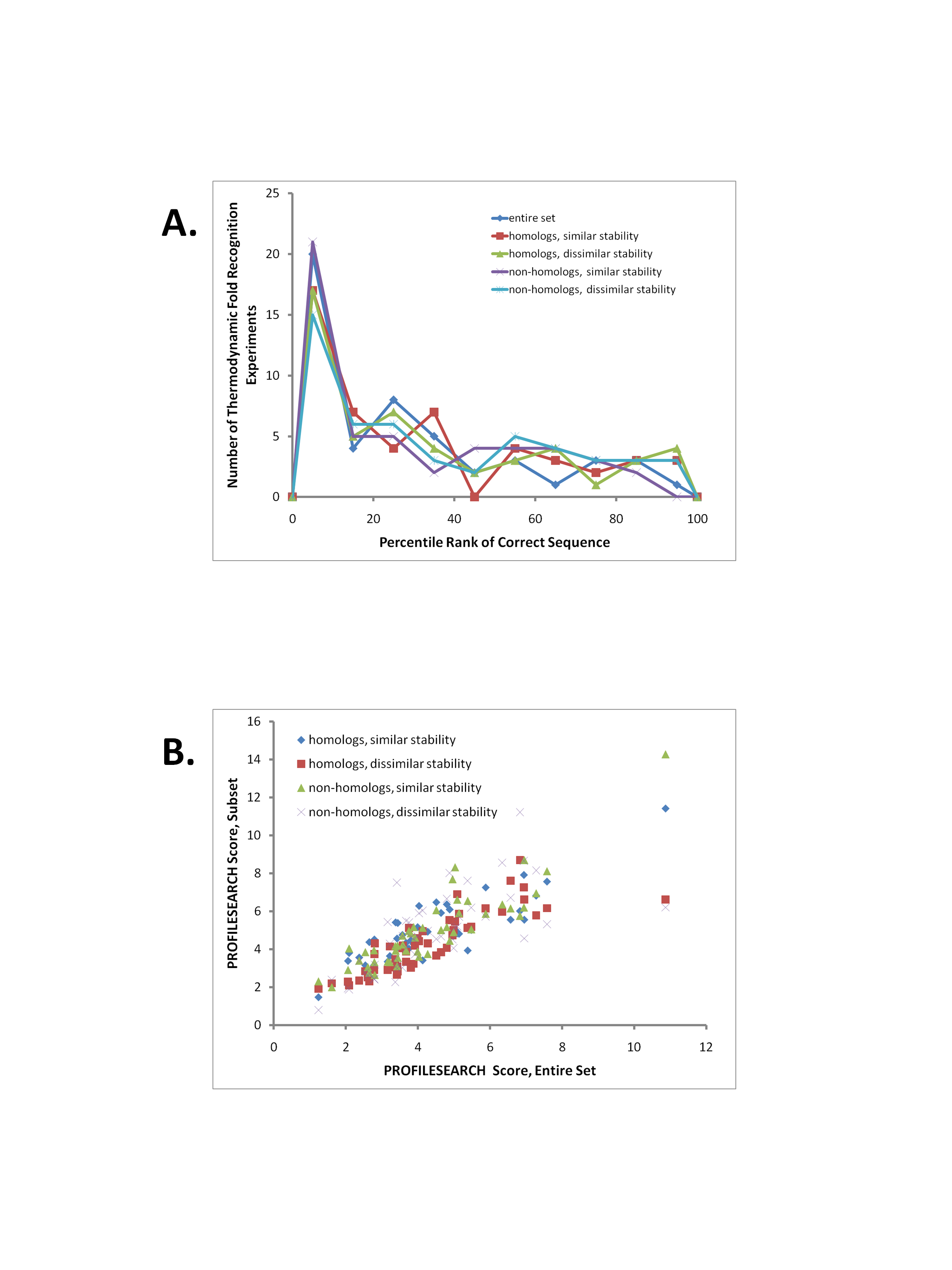

Supplement: Figure S3 — Thermodynamic fold recognition results for fifty randomly chosen query proteins using information derived from subsets of residue positions. Subsets of residue positions were constructed from regions of overlap, as described in the text. A. Distributions of percentile rank for fold-recognition Z-scores between a thermodynamically defined query and its correct amino acid sequence. Successful fold recognition experiments exhibit lower percentile ranks. Distributions of percentile ranks are similar regardless of the subset source of thermodynamic information. B. Correlation of fold recognition raw scores between experiments using different subset sources of thermodynamic information. Scores are similar (visibly correlated) regardless of the subset source of thermodynamic information. (1.04 MB TIF) [file pcbi.1000722.s004.tif]
